# Detection of mutations associated with artemisinin resistance at k13-propeller gene and a near complete return of chloroquine susceptible falciparum malaria in Southeast of Tanzania

**DOI:** 10.1038/s41598-020-60549-7

**Published:** 2020-02-26

**Authors:** George M. Bwire, Billy Ngasala, Wigilya P. Mikomangwa, Manase Kilonzi, Appolinary A. R. Kamuhabwa

**Affiliations:** 1 Department of Pharmaceutical Microbiology, Muhimbili University of Health and Allied Sciences, P.O. Box 65013 Dar es Salaam, Tanzania; 2 Department of Parasitology and Medical Entomology, Muhimbili University of Health and Allied Sciences, P.O. Box 65001 Dar es Salaam, Tanzania; 3 Department of Clinical Pharmacy and Pharmacology, Muhimbili University of Health and Allied Sciences, P.O. Box 65013 Dar es Salaam, Tanzania

**Keywords:** Infectious diseases, Public health, Parasite genetics

## Abstract

In Tanzania, chloroquine was replaced by sulphadoxine- pyrimethamine (SP) as a first-line for treatment of uncomplicated malaria. Due to high resistance in malaria parasites, SP lasted for only 5 years and by the end of 2006 it was replaced with the current artemisinin combination therapy. We therefore, set a study to determine the current genotypic mutations associated with *Plasmodium falciparum* resistance to artemisinin, partner drugs and chloroquine. Parasites DNA were extracted from dried blood spots collected by finger-prick from Tanzanian malaria infected patients. DNA were sequenced using MiSeq then genotypes were translated into drug resistance haplotypes at Wellcome Sanger Institute, UK. About 422 samples were successful sequenced for *K13* gene (marker for artemisinin resistance), the wild type (WT) was found in 391 samples (92.7%) whereby 31 samples (7.3%) had mutations in *K13* gene. Of 31 samples with mutations, one sample had R561H, a mutation that has been associated with delayed parasite clearance in Southeast Asia, another sample had A578S, a mutation not associated with artemisinin whilst 29 samples had *K13* novel mutations. There were no mutations in *PGB*, *EXO, P23_BP* and *PfMDR1* at position 86 and 1246 (markers for resistance in artemisinin partner drugs) but 270 samples (60.4%) had mutations at *PfMDR1* Y184F. Additionally, genotyped *PfCRT* at positions 72–76 (major predictors for chroquine resistance), found WT gene in 443 out of 444 samples (99.8%). In conclusion, this study found mutations in *K13*-propeller gene and high prevalence of chloroquine susceptible *P. falciparum* in Southeast of Tanzania.

## Introduction

Artemisinin-based combination therapy (ACT) is recommended by World Health Organization (WHO) to its partner states^1,2^ as the first and second-line treatment for uncomplicated *Plasmodium falciparum* malaria as well as chloroquine-resistant *Plasmodium vivax*^3^. In Tanzania chloroquine (CQ) was replaced by sulphadoxine- pyrimethamine (SP) as first-line treatment and amodiaquine as second-line for uncomplicated malaria, due to high resistance SP lasted for only five years and by the end of 2006 it was replaced with the current ACT^4^. Reversibly, an extended use of artemisinin (ART)-based combination therapy in malaria control and elimination programs has resulted to an emergence of *P. falciparum* resistant to ART derivatives in Southeast Asia^5^. The risk of ART-resistant parasites reported to spread from western Cambodia to the Greater Mekong Subregion and to Africa^6^. This is an urgent concern for global health^7^. The spread of resistant *P. falciparum* to previous first-line treatment of malaria (chloroquine and sulfadoxine-pyrimethamine) in nearly all endemic countries originated in almost similar fashion^8^.

The discovery of mutations in the propeller domain of the kelch (*K13)* gene were marked as candidate molecular markers and has remained to be the key predictor of ART resistance^9^. Several mutations at *K13* are therefore associated with ART resistance^10^, i.e. *K13* M476I mutation was first investigated in Tanzanian F32 parasites that were exposed *in vitro* to escalating concentrations of ART for more than 5 years^11,12^. In addition to that genomic analysis of Cambodian isolates identified four prevalent *K13* mutations (Y493H, R539T, I543T and C580Y) that were associated with ART resistance^12,13^. In this regard, the list of *K13* has kept on increasing and regularly updated by World Health Organization (WHO)^3^. Additionally, evidence from research reported parasite genetic background (PGB), is the mutations that allowed the emergence of *K13* mutations, these mutations include; V127M and D128Y/H in the *PfARPS10* (PF3D7_1460900) protein, D193Y in *ferredoxin* (*PfFD*, PF3D7_1318100), N326S and I356T in *PfCRT* (PF3D7_0709000), and T484I in *PfMDR2* (PF3D7_1447900) and they are expressed as concatenated haplotype form (VDDNIT) as a reference allele (wild type)^14^.

Moreover, *P. falciparum* multi-drug resistance gene 1 (*PfMDR1*, PF3D7_0523000) and particularly, single nucleotide polymorphisms (SNPs) resulting in an amino acid change in codons 86 (N86Y), 184 (Y184F), and 1246 (D1246Y) have been associated with changes in parasite susceptibility to various drugs, including ACT. Mutations at position 86 and 1246 have been associated with parasite resistance to CQ and amodiaquine^15^ while mutations at positions 86, 184, and 1246 increase susceptibility of mefloquine and lumefantrine^16^. Genome‐wide association study (GWAS), a single nucleotide polymorphism (SNP) in a putative *exonuclease* gene (*PfEXO*, Pf3D7_1362500) was associated with an increased tolerance of piperaquine.

On the other hand, gene amplification of a section of chromosome 14 involving the genes plasmepsin 2 and plasmepsin 3, P2/3 breakpoint (*P23_BP*) has been associated with an increased resistance to piperaquine^17^. Additionally, CQ resistance transporter (*PfCRT*) and *PfMDR1* both located on the food vacuole of the parasite involved in CQ resistance^18^. However, the CQ transporter *PfCRT* is a stronger predictor of CQ resistance than *PfMDR1*^19^. Nevertheless, in areas where usage has been strictly regulated, withdrawal of CQ have resulted in dramatic decreases in the prevalence of CQ-resistant parasites^4,20,21^.

Therefore, it was necessary to conduct a molecular surveillance of gene mutations associated with *P. falciparum* resistance to artemisinin, partner drugs and chloroquine, one decade since ART-based combination therapy was introduced as a first line treatment and 18 years after CQ withdrawal for treatment of uncomplicated malaria in Tanzania^4^.

## Results

### *kelch13* (*K13*) propeller polymorphisms

A total of 489 samples were genotyped but 67 (13.7%) could not be detected/missing genotypes. Of 422 successful sequenced samples, wild type (WT) *K13* gene was found in 391 samples (92.7%) whereby 31 samples (7.3%) had mutations in *K13* gene. Of those mutations, one sample had R561H, a mutation that has been associated with delayed parasite clearance, another sample had A578S, a mutation not associated with clinical or *in vitro* resistance to artemisinin whilst 24 nonsynonymous mutations are not yet listed (uncharacterized) in WHO artemisinin resistance report of 2018 (Table 1).

**Table 1:** Frequency of *K13* propeller mutations.

*kelch13* mutation (*K13*)	Frequency n (%)	WHO classification of 2018			
		Validated	Not Associated	Candidate	Not yet classified
WT	391 (92.7)	NA	NA	NA	NA
A359T	1 (0.2)				√
A427S	1 (0.2)				√
C469C	4 (0.9)				√
G538G	1 (0.2)				√
I354V	1 (0.2)				√
I526I	1 (0.2)				√
P417P	1 (0.2)				√
S624S	1 (0.2)				√
V487V	1 (0.2)				√
WT A486A	1 (0.2)				√
WT C469C	1 (0.2)				√
WT C473C	1 (0.2)				√
WT G449C	1 (0.2)				√
WT D648G	1 (0.2)				√
WT G638R	1 (0.2)				√
WT P413P	1 (0.2)				√
WT P417P	1 (0.2)				√
WT R471S	1 (0.2)				√
WT R561H	1 (0.2)	√			
WT S485N	1 (0.2)				√
WT S624S	2 (0.4)				√
WT V487V	2 (0.4)				√
WT V666V	1 (0.2)				√
WT A578S WT W565C	1 (0.2)		*		√
WT S624S WT F439S WT P417P	1 (0.2)				√
Total	422	NA	NA	NA	NA

NA: Not Applicable; ^√^Indicates the appropriate classification category, *within a clone (WT A578S WT W565C), A578S has been classified by WHO as not associated with ART resistance.

### Prevalence *PGB*, *EXO/P23_BP* and *PfMDR1* mutations

Generally, 489 samples were genotyped but the total number per every marker differed based on the number genotypes could be detected. All genotypes for *PGB* and *EXO/P23_BP*, markers for ART and piperaquine resistance respectively were WT. There were 270 samples (60%) with mutations at *PfMDR1* Y184F; a marker for lumefantrine, amodiaquine and mefloquine drug (Table 2).

**Table 2:** Frequency of *PGB*, *EXO/P23_BP* and *PfMDR1* mutations.

Drug	Gene	Gene status	
		WT, n (%)	Mutation, n (%)
Artemisinin	*PGB*	447 (100)	0
Piperaquine	*EXO*	446 (100)	0
	*P23_BP*	336 (100)	0
Lumefantrine Mefloquine Amodiaquine Chloroquine	*MDR1* N86Y	436 (100)	0
	*MDR1* Y184F	177 (39.6)	270 (60.4)
	*MDR1* D1246Y	447 (100)	0

Mutation at MDR1 86 is associated with chloroquine resistance while limited evidences associate mutations at 86 and 1246 with lumefantrine, mefloquine and amodiaquine resistance.

### Prevalence of *PfCRT* polymorphisms

Of 443 genotyped samples and analyzed for the *PfCRT* 76, 442 samples (99.8%) contained the wild type (WT)/susceptible (K76) while only one (0.2%) threonine (76T) was detected. The *PfCRT* haplotypes at positions 72–76, CMNVK were detected in 442 samples (99.8%). while the resistant haplotype CVIET was detected in only one (0.2%) samples (Table 3). The *PfMDR1* mutation at position N86Y is the first in the 3 amino‐acid haplotype (NYD) which enhances resistance to CQ was not detected in the all samples (100%) (Table 2).

**Table 3:** Frequency of *PfCRT* polymorphisms.

Gene (AA position)	Resistant n (%)	Heterozygous n (%)	Susceptible n (%)
*PfCRT* (72, 73, 74, 75, 76)	1 (0.2)	—	442 (99.8)
*PfCRT* (K76T)	1 (0.2)	—	442 (99.8)
*PfCRT* (93)	0	—	444 (100)
*PfCRT* (97)	0	—	444 (100)
*PfCRT* (218)	0	—	445 (100)
*PfCRT* (220)	1 (0.2)	3 (0.6)	441 (99.1)
*PfCRT* (271)	1 (0.2)	2 (0.5)	440 (99.3)
*PfCRT* (333)	0	—	445 (100)
*PfCRT* (353)	0	—	443 (100)
*PfCRT* (371)	1 (0.2)	2 (0.5)	442 (99.3)

AA: Amino Acid; -:Not detected.

Note: Differences in the total number of genotypes were due to missing genotypes in some samples. If two alleles were detected, then it was assigned to heterozygous call category.

## Discussion

To the best of authors’ knowledge this is the first study to report mutations at *K13*-propeller, associated with ART resistance from the southeast of Tanzania. We report resistance patterns, one decade since ART-based combination therapy was introduced as the first and second line treatment of malaria whilst 18 years after CQ withdrawal for treatment of uncomplicated malaria in Tanzania. Generally, this study found a prevalence of 7.3% for *K13* mutations, these mutations contained those which are found in WHO list^3^, and those reported elsewhere^10^ and undocumented non-synonymous *K13* mutations. Prevalence obtained in this study was higher than the one reported the polymorphisms of *P. falciparum K13*-propeller gene among migrant workers returning to Henan Province, China from Africa, the study found the frequency of the *K13*-propeller 6.50% in Central Africa, followed by East Africa (5.26%), West Africa (4.55%) and South Africa (4.55%)^6^ and study by Kamau *et al*., 2014^22^ reported that, allele frequencies of *K13*-propeller polymorphisms in *P. falciparum* parasites from sub-Saharan Africa ranged between 1% and 3%. In contrary to that, the recent findings from Uganda^23^, Kenya^24^ as well as Tanzania^25^ reported no evidence of *K13* mutations. The differences in study periods between the studies could be the cause of the observed discrepancies in prevalence.

*K13* mutations at position R561H and A578S, are two mutations previously described by WHO as validated^3^ and not associated^26^ with ART resistance, respectively. Surprisingly, both two mutations were detected as recombinant which contained WT gene, i.e. WT R561H and WT A578S WT W565C. These mutations especially, the validated mutation R561H cause a delayed parasite clearance^10,27^. Mutation in *K13* gene has also been reported from the study conducted in southern Rwanda^28^. More importantly, this study documented 24 *K13* mutations which currently don’t form part of WHO ART resistance markers list of 2018^3^. On the other hand, all genotypes for PGB and EXO/P23_BP, markers for ART and piperaquine resistance respectively found no mutation.

High prevalence (60.4%) *PfMDR1*Y184F mutations was detected from Tanzanian samples. These finding are similar from the study conducted in Equatorial Guinea which found high prevalence of *PfMDR1* Y184F mutations in *P. falciparum* isolates. Nevertheless, there are limited evidences which associated the *PfMDR1*Y184F mutation with lumefantrine and mefloquine^16^ susceptibility. Furthermore, the study reported that *PfMDR1* at amino acids 86 and 184, demonstrate resistance to the ACT partner drug amodiaquine and the former first-line agent CQ. In contrast, N86Y increases parasite susceptibility to the partner drugs lumefantrine and mefloquine, and the active artemisinin metabolite dihydroartemisinin. The *PfMDR1* N86Y plus Y184F isoform moderately reduces piperaquine potency in strains expressing an Asian/African variant of the CQ resistance transporter *PfCRT*^16^. On the other hand, the current findings suggest that CQ-susceptible *P. falciparum* parasites have reemerged and are now predominant in Tanzania (sub-Saharan Africa) where CQ was withdrawn in 2001. These findings were similar from the recent study conducted in Zambia^29^, the neighboring country to southwest. In both two countries CQ were withdrawn as a first line treatment in 2001^4^ and 2003^21^ in Tanzania and Zambia, respectively. These findings were contrary to the country where high levels of CQ resistance have persisted due to incomplete withdrawal of CQ^30^. The study of 2019, conducted in Nigeria revealed a high prevalence of *PfCRT* mutant genotypes and haplotypes and low frequency of *PfMDR1* mutant genotypes, 11 years after the switch in malaria treatment policy from CQ to artemisinin combination therapy (ACT) in Nnewi, Nigeria. The study suggested that continual circulation and spread of CQ-resistant *P. falciparum* parasites in the study area due to the continued use of unrecommended CQ^30^.

Moreover, this study found a complete deletion of *PfMDR1* mutation in all samples at position N86Y, a mutation which is associated with an enhanced resistance to chloroquine. These findings are in line with those reported in a study conducted in Zambian^29^. Another study conducted in Malawi reported a slower decline in prevalence of mutations in *PfMDR1* than *PfCRT* suggesting that *PfMDR1* mutations may be less deleterious to parasite fitness than are *PfCRT* mutations^31^. However, mutations in *PfMDR1* by themselves are insufficient to confer CQ-resistance^32^ also the combination of *PfMDR1* mutations and *PfCRT* mutations, provided no added advantage to CQ treatment failure than *PfCRT* mutations alone^33^. Furthermore, *PfMDR1* mutations do not add to the predictive value of *PfCRT* mutations for CQ treatment failure^34^.

Since the design of the current study was cross sectional, there was a limitation in establishing the association between the molecular resistance markers and clinical/treatment outcomes of patients. However, the relationship between malaria treatment outcomes and resistance markers have been well described elsewhere^3,10^.

In conclusion, *K13*-propeller mutations associated with artemisinin resistance were found in Tanzanian samples. Mutations included *K13* R561H and *K13* A578S haplotypes validated to cause artemisinin resistance and not associated with artemisinin resistance, respectively. Twenty four *K13* non-synonymous mutations not yet listed by WHO (unclassified) are reported. Further phenotypic studies are warranted to investigate the unclassified *K13* mutations. Additionally, the return of chloroquine-susceptible *P. falciparum* malaria, 18 years after the removal of chloroquine drug pressure in Tanzania is documented. In this regard, chloroquine may be considered for malaria prevention, i.e. sickle cell disease children or the reintroduction in future, ideally in combination with other antimalarial drugs, especially in areas where disappearance of chloroquine resistance is evident while safe and affordable alternatives antimalarials are limited.

## Materials and Methods

### Study design, area, period and population

**Figure 1:**
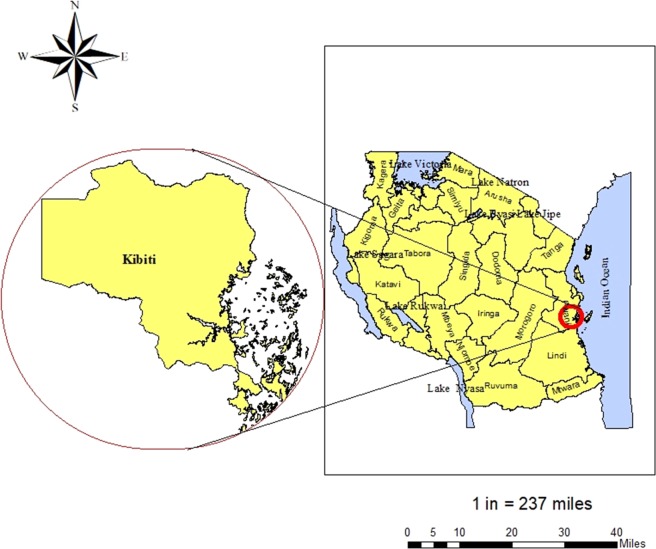
A map of the study site. Left is the map of Kibiti District while on the right side is the map of Tanzania, located in Eastern Africa. Tanzania is bordered by the Indian Ocean, Kenya and Uganda to the north, Rwanda, Democratic Republic of the Congo, and Burundi to the east, and Zambia, Malawi, and Mozambique to the south (not shown on the map). The study site map was originally generated using ArcGIS software version 10.7.1 (https://www.esri.uconn.edu/software/arcgis-student/).

Surveillance of molecular markers for ART and partner drugs (piperaquine, lumefantrine, amodiaquine, mefloquine and chloroquine) resistance was conducted between April and August 2019 at Kibiti Health Center (KHC), Kibiti District, Tanzania (Fig. 1). Kibiti District is found along the Indian ocean^35^ and has malaria prevalence of 10.2%^36^ where *P. falciparum* is responsible for more than 95% of all malaria cases^37^. Patients attending clinic at Kibiti Health Center (KHC) who presented with symptoms suggestive of malaria infection were recruited in the study. The symptoms such as fever, general body weakness and headache were confirmed by the attending physician^38^. Patients screened for malaria and those who tested positive using CareStart Malaria HRP2/pLDH test (Access Bio, Ethiopia) were requested to participate. Then positive samples by rapid tests were subjected to blood smear (BS) microscopy for confirmation. A total of 489 dried blood samples (DBS) from patients tested positive with BS microscopy were subjected to DNA extraction and genotyping.

#### DBS preparation

DBS were prepared according to MalariaGEN SpotMalaria, DBS collection protocol^39^. A sterilized patient’s finger was pricked to allow blood drops where four blood spots from each patient were prepared, two on each paper card. The blood spots were allowed to air dry and placed in the desiccant sachet for storage.

#### DNA extraction

DNA from the DBS was extracted following QIAamp DNA Investigator Kit for isolation of total DNA from filter papers (Qiagen, Valencia, CA, USA) and as previously described by Oyola *et al*.^40^.

### Genotyping of antimalarial resistance markers

Molecular genotyping of ART, partner drugs and chloroquine (*K13, PGB, EXO, P23_BP, MDR1 and CRT*) were performed by Wellcome Sanger Institute, UK.

Briefly, targets for genotyping were identified and multiplex PCR primers were designed using a modified version of the mPrimer software^41^ and the exact design of the primer sequences will be described elsewhere (Goncalves, manuscript in preparation). Primers were designed to amplify products between 190–250 bp and were combined into 3 pools. A two-step protocol was used to first amplify the target regions of the parasite genome, followed by a second PCR to incorporate sequencing and multiplexing adapters. Batched samples (384) were sequenced in a single MiSeq lane combining all PCR products. Samples were de-plexed using the multiplexing adapters and individual CRAM files were aligned to a modified amplicon reference genome. Genotyping was done using bcftools as well as custom scripts to filter and translate genotypes into drug resistance haplotypes.

#### Statistical analysis

Laboratory information on Microsoft Excel Sheet (Redmond, WA) were exported directly to Statistical Package for Social Sciences version 25 (SPSS Software, Chicago Inc., USA) for data analysis. Genotypes were expressed as frequencies and percentages.

### Ethics approval and consent to participate

Ethical approval to conduct this study was sought from Muhimbili University of Health and Allied Sciences Ethical Review Board (Ref. DA.282/298/01A.C/) and National Institute for Medical Research (Ref. NIMR/HQ/R.8A/Vol.IX/3107). Permission to conduct the study at KHC was obtained from both Kibiti District Medical Officer and KHC Medical Officer In-charge. Written informed consent after explaining the purpose of the study was requested before enrollment of participants. Additionally, all methods were carried out in accordance with relevant guidelines, regulations and good laboratory practice.

## Data Availability

The datasets generated and/or analysed during the current study are available from the corresponding author on reasonable request.
